# Supplementary material for: Patterns of Intron Gain and Loss in Fungi
Source: PLoS Biol. 2004 Nov 30;2(12):e422. doi: 10.1371/journal.pbio.0020422 (PMC532390; doi:10.1371/journal.pbio.0020422)
Supplement: Table S1 — Also available at http://genes.mit.edu/NielsenEtAl/. (4.3 MB ZIP). [file pbio.0020422.st001.zip › NielsenEtAl/html/945.html]

AN8224.1.NCU08894.1.MG05956.1.FG04582.1


```
 CLUSTAL W (1.82) Multiple Sequence Alignments - Introns Inserted


Sequence 1: NCU08894.1	636 aa
Sequence 2: MG05956.1	635 aa
Sequence 3: FG04582.1	624 aa
Sequence 4: AN8224.1	677 aa
Alignment Length: 734 aa
Number Identitical Residues: 224 aa
Alignment Score (without introns) 13565


MG05956.1 	------------------------------------------------------------
NCU08894.1	------------------------------------------------------------
FG04582.1 	------------------------------------------------------------
AN8224.1  	MSQFQLTLATKANQAALLPILLVATSVNEARPAPVITINYEDATVLPRGDKEIVEFTAAN
          	 :. . : ::.:..::  .    ::: ..: .:.  : . ..::  . ....  . :::.

MG05956.1 	--------------------------M~KAEELETIAPALSGDFRSIEGHLTNLDKHLTL
NCU08894.1	--------------------------M~KLDELKPLEALLTADFRQIEPRLQALDKHLIL
FG04582.1 	--------------------------M~TFDAEK----IGAADFKSLGPLVAELDKHLTL
AN8224.1  	GTLVYGSAAFKELLLSFPYLNSKEEKL0ENEWLAQLEDFASLDFKAIEPKLQQLNNHLIL
          	.:   .::: ..   : .  .:....:   :         : **: :   :  *::** *

MG05956.1 	RTYLNGTQLGDIDSKIWTSLATNRAAIAFIRRGVLANLTRWFIFVEQNHPEIQGEVKKAA
NCU08894.1	RTYLDGYTLGDIDTKIWLALRGNRAAVSFIKRGSLANLARWFNYIEENHPEIQAEIKAKD
FG04582.1 	RTYLNGYTLSEEDGKIWTALRTNKVAIGLVRKGAYTNITRWFKLIEEAHPEFNEKLN---
AN8224.1  	RSFVVGYALSTADIALWGALRGNRVAIGAIRKGTLINLTRWFNFLEELCPWAVAALEALN
          	*::: *  *.  *  :* :*  *:.*:. :::*   *::***  :*:  *     ::   

MG05956.1 	AAQKAKVAAASRAG~GNFNLALQDADKGVVTRFLPEPS2GYLHIGHAKAALLSDYFAHQA
NCU08894.1	AAAKAKVAAASKAG~ASYALALQNADQGVVTRFLPEPS~GYLHIGHAKAALLSDYFAHQA
FG04582.1 	-AGKEKRS----GG~ANYNIGLSNTENGVVTRFPPEPS2GYLHIGHAKAALLNDYFAKTS
AN8224.1  	ASAKEKRAAKAEGR2GRH----------------PFPS~GAFWIP-----------PHRT
          	:: * * :: : .  . .                * ** * : *            .: :

MG05956.1 	YNG--TLRLRLDDTNPSKEKE~EYQDAIIEDLALMGIKADKFSYTSDYFQYLYEMCIRLI
NCU08894.1	YKG--QLRLRLDDTNPSKEKQ~EYQDAIIEDLALMGIKPDTVTYTSDYFDYLYDMCVRMI
FG04582.1 	PDGNGKLIVRFDDTNPAKEKQ~EFEDAILHDLELMDIKYHKVTHSSDYFQEIYDLTEKMI
AN8224.1  	RQG------RASER-LLRPRE0EFQDAILEDLALMGIKPDKTSYSSDYFDELQQYAVQII
          	 .*      * .:    : :: *::***:.** **.** .. :::****: : :   ::*

MG05956.1 	KEGNAYADDTDQETMKKERWDGIASKHRDMSIEETLRVF-EEMKKGSDEGKRYCIRAKLS
NCU08894.1	KEGHAYADDTDQDTMRDQRWKGIASARRDRSVEENLRIFTEEMKNGTEEGLKNCIRAKLS
FG04582.1 	QDGNAYADDTDPEVQSADRKNRLASKRRDRPAEESLAMF-REMKAGTDLGRKHCIRARIA
AN8224.1  	KDGNAYADDTDKETMASQRMNGEPSKRRDATVEENLARF-EEMTKGTEEGARWCIRAKMS
          	::*:******* :.   :* .  .* :** . **.*  * .**. *:: * : ****:::

MG05956.1 	VDNPNKALRDPVIYRC-----NDE-PHHRTGTTWKMY~PMYDFACPVVDSYEGVTHALRS
NCU08894.1	VDNPNKALRDPVIYRC-----NIETPHHRTGTKWKMY~PMYDFACPVVDSHEGVTHALRS
FG04582.1 	FDSSNGSMRDPVIFRFPNWKGEEPAPHHRTGWTWNIY0PTYDLVVPILDSIEGVTHALRT
AN8224.1  	VDNPNKAMRDPVIYRC------NPAPHHRTGTKYKAY~PTYDFCCPIIDSLEGVTHALRT
          	.*..* ::*****:*         :****** .:: * * **:  *::** ********:

MG05956.1 	TEYTDRNPQYQWFLDTLKLRQVHMWDFARMNFIRTFLSKRKLAKLVDTGRVWGWDDPRMP
NCU08894.1	TEYTDRNPQYQWFIDTLKLRQVYMWDFARMNFIRTFLSKRKLAKLVDTGKVWGWDDPRMP
FG04582.1 	TEYADRNEQYHWFLKALNLRQVHLWDFARINFIQTFLSKRKLTKVVDTGRVSGWDDPRMP
AN8224.1  	IEYRDRNPQYQWFLDTLKLRNVQIWDFARMNFVRTLLSKRKLTKLVEQGVVDGWSDPRFP
          	 ** *** **:**:.:*:**:* :*****:**::*:******:*:*: * * **.***:*

MG05956.1 	TIRGVRRRGMTVPALRDFIIKQGPSRNVTSMDWTVFWAANKKEIDPVAARHTALSQKSPV
NCU08894.1	TIRGVRRRGMTIPALRDFILKQGPSRNVVTMDWTNFWASNKKEIDPIAPRHTAITKKDAV
FG04582.1 	TVRGIIRRGLTVPALREFMLKQGPSRNVVTMDWTTIWAINKRMLDPVVPRYMAIEEKDAV
AN8224.1  	TIRGIRRRGMTIPALREFILKQGPSRNIVNLDWTSFWATNKKYIDPVAPRHTAVLKKDAV
          	*:**: ***:*:****:*::*******:..:*** :** **: :**:..*: *: :*..*

MG05956.1 	KVKITGKDAPASPHSVEKPKHPKNKDVGVKQVAFSDEILLEQEDVALMKPGEEITLMAWG
NCU08894.1	KVVVKGADAPAEPVKQEKPKHPKNKEVGTKQVTFANELIMDQADAKSFKDGEEITIMGWG
FG04582.1 	TVTITG--GPEKSYKEDRPKHVKNPDVGTKQVTFGPKLLLDQVDVASFDDNEEITLMSWG
AN8224.1  	KATIKG--GPDSPYTQDKPKHNKNPDVGTKKVTYSSSILFDQEDAKSFKQDEEITLMNWG
          	.. :.*  .* .. . ::*** ** :**.*:*::. .::::* *.  :. .****:* **

MG05956.1 	NAIVKDIST-GTPIP-TMTVELNLAGDVKTTEKKVTWLSNQGQKLIPAELWDFDYLITKD
NCU08894.1	NGFVRNIDTSAEVIP-AFEIDLNLAGDVKSTEKKVTWLASKGQTLVPAELWDFDYLITKD
FG04582.1 	NAIVRGINKTASPIN-ELNLELHLAGDFKTTSKKVHWLAADPENLVKAELWEFGSLITKD
AN8224.1  	NAFVRKIVTDASGVVTELELDLNEKGDFKKTEKKVTWLSTD-QDLVPVDLVDFDYLLNKD
          	*.:*: * . .  : : : ::*:  **.*.*.*** **: . : *: .:* :*. *:.**

MG05956.1 	KLEEDDDLEKFLTPVTEFMEPAWCGADAETLKKDDVVQLERRGYYRVDKGLGDWKDGEEG
NCU08894.1	VLQEEDNMEDFLNPVTETMEEAWCDEAAASLKKDDIIQLERRGYYRVDKGLNDWKEGEE-
FG04582.1 	TLEKDDNLDDFLNDNSATLTNALVDASIAELKENDFLQLERKGYYRVDKALGQGPDGRA-
AN8224.1  	SMQEEDSLEDVLNPKTEFHEDAVADSNVALLAEGDIIQFERKGYYRLDKVASP---GKP-
          	 ::::*.::..*.  :     *  .     * :.*.:*:**:****:**  .    *.  

MG05956.1 	EKGKRIVFFNIPTG~KTK--
NCU08894.1	---KKLVLFCIPTG~KTGSK
FG04582.1 	------VLFKVPTG~GQKG-
AN8224.1  	-----AVFFNIPSD1E----
          	      *:* :*:.
```
